# Supplementary material for: Recruitment of upper-limb motoneurons with epidural electrical stimulation of the cervical spinal cord
Source: Nat Commun. 2021 Jan 19;12:435. doi: 10.1038/s41467-020-20703-1 (PMC7815834; doi:10.1038/s41467-020-20703-1)
Supplement: Supplementary file 1 — Supplementary Information [file 41467_2020_20703_MOESM1_ESM.pdf]

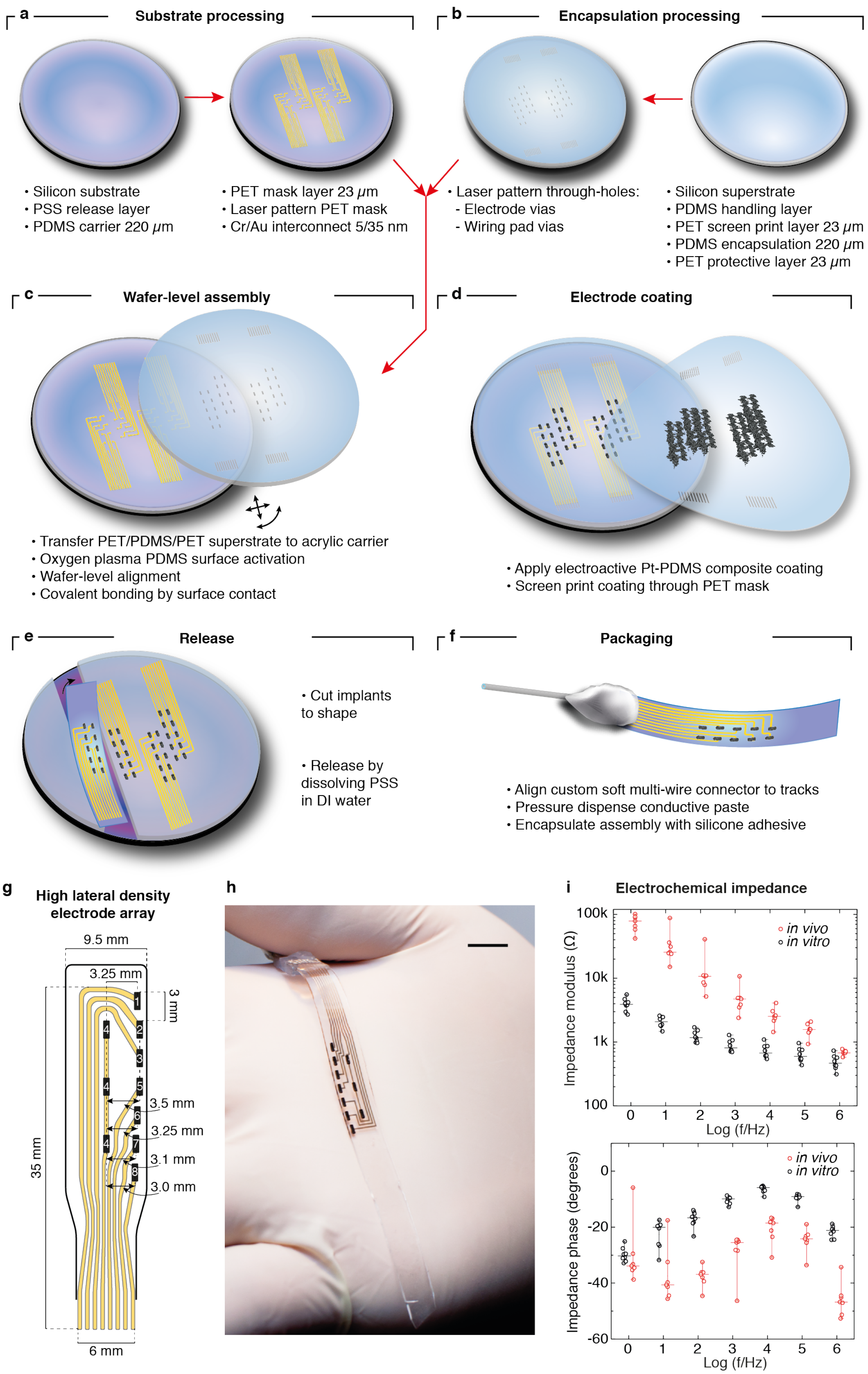
a
Substrate processing
• Silicon substrate
• PSS release layer
• PDMS carrier 220 μm
• PET mask layer 23 μm
• Laser pattern PET mask
• Cr/Au interconnect 5/35 nm
b
Encapsulation processing
• Laser pattern through-holes:
- Electrode vias
- Wiring pad vias
• Silicon superstrate
• PDMS handling layer
• PET screen print layer 23 μm
• PDMS encapsulation 220 μm
• PET protective layer 23 μm
c
Wafer-level assembly
• Transfer PET/PDMS/PET superstrate to acrylic carrier
• Oxygen plasma PDMS surface activation
• Wafer-level alignment
• Covalent bonding by surface contact
d
Electrode coating
• Apply electroactive Pt-PDMS composite coating
• Screen print coating through PET mask
e
Release
• Cut implants to shape
• Release by dissolving PSS in DI water
f
Packaging
• Align custom soft multi-wire connector to tracks
• Pressure dispense conductive paste
• Encapsulate assembly with silicone adhesive
g
High lateral density electrode array
9.5 mm
3.25 mm
3 mm
35 mm
3.5 mm
3.25 mm
3.1 mm
3.0 mm
6 mm
h
i
Electrochemical impedance
Impedance modulus (Ω)
100k
10k
1k
100
in vivo
in vitro
Log (f/Hz)
Impedance phase (degrees)
0
-20
-40
-60
0 1 2 3 4 5 6

Supplementary Figure 1. Electrode array technology. a-f Process flow for the fabrication of soft implants on silicon wafers. **a** Substrate processing with the deposition of the stretchable interconnect. **b** Encapsulation processing with the laser micromachining of through-vias. **c** Assembly by covalent bonding of the PDMS substrate and encapsulation. **d** Screen printing of the electrode coating. **e** Implant release from the silicon wafer. **f** Wiring and packaging of the implant. Electrode arrays for cervical neuromodulation. **g** Custom layout of an electrode array with 7 lateral and 1 medial (split in three pieces) electrode contacts (design 2) tailored to the monkey cervical spinal cord. **e** Photograph of a cervical soft electrode array with lateral electrode placement. *Scale bar:* 1 cm. **f** Electrochemical impedance spectrum acquired on an electrode array implanted in a monkey. Data points are shown at frequencies $f_i = 10^i$, where i = 0, 1, ..., 6. *Black data points:* data *in vitro*, n = 8 electrodes. *Red data points:* data *in vivo*, n = 7 electrodes. *Horizontal bars:* means. *Error bars:* minima-maxima.

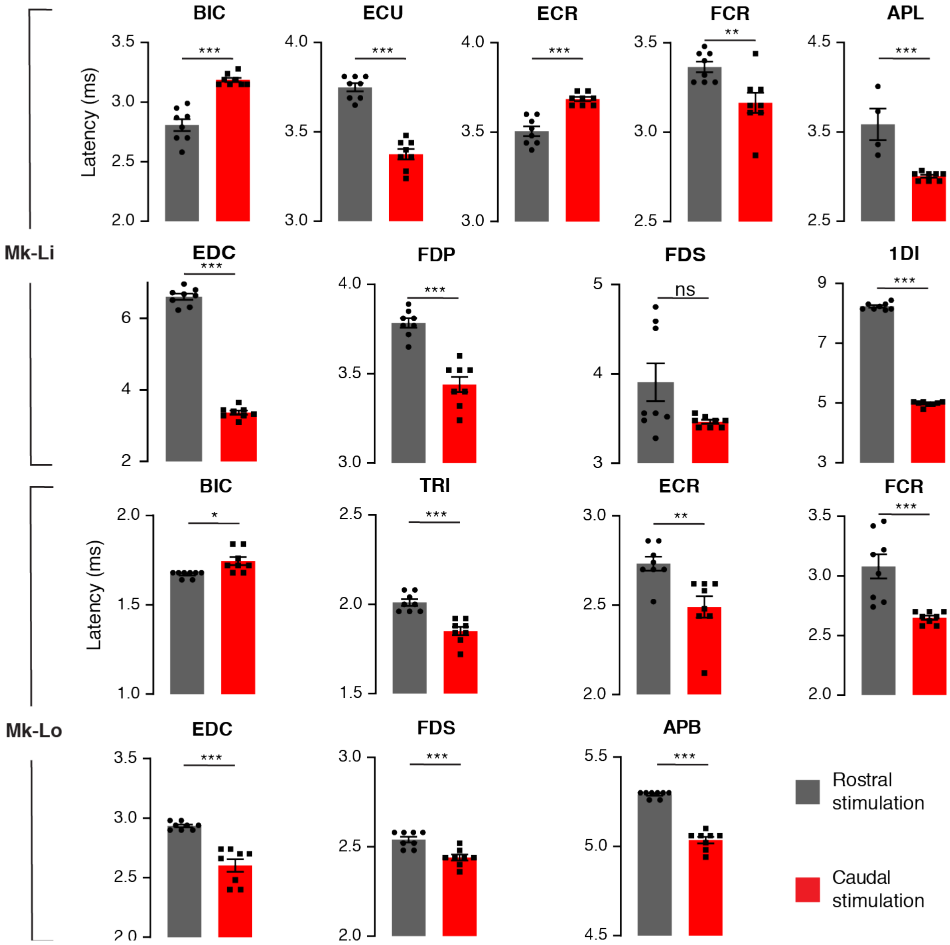

Supplementary Figure 2. Latency analysis of muscular responses elicited by medial stimulation. *Top:* latencies of muscular responses recorded in monkey Mk-Li. *Bottom:* latencies of muscular responses recorded in monkey Mk-Lo. For both, all muscles candidate for the analysis were included, and the latencies of 8 responses evoked at stimulation intensities close to motor threshold for one rostral medial and one medial caudal electrode contacts were retained (see **Methods**). *Black-filled circles and squares:* data points. *Bars and whiskers:* means ± SEMs across the 8 data points. *Statistics:* two-sided unpaired *t*-tests, ** $p<0.01$; *** $p<0.001$ (see **Methods**). BIC: biceps, ECU: extensor carpi ulnaris, ECR: extensor carpi radialis, FCR: flexor carpi radialis, APL: TRI: triceps, FDS: flexor digitorum superficialis, EDC: extensor digitorum communis, APB: abductor pollicis brevis.

Table 1. Estimated characteristics of the connectivity between Ia-afferents and motoneurons in 8 upper-limb muscles of the monkey. DEL: deltoid. BIC: biceps. TRI: triceps. EDC: extensor digitorum communis. ECR: extensor carpi radialis. FDS: flexor digitorum superficialis. FCR: flexor carpi radialis. APB: abductor policis brevis.

Muscle	Ia-fiber headcount	Mean number of synapses per motoneuron	Synaptic conductance [pS]	Total conductance per motoneuron [pS]
DEL	68	578	9.625	5560
BIC	119	1036	5.0	5180
TRI	193	1681	3.375	5675
EDC	82	699	7.625	5330
ECR	65	555	10.5	5830
FDS	132	1157	5.75	6650
FCR	48	408	15.375	6275
APB	30	250	28.5	7125

Table 2. Identification information, characteristics, type of procedure performed, and license numbers of the monkeys involved in the study.

Animal ID	Authorization number	Sex	Age	Weight	Procedure
Mk-Ca	2014_42E_ FR	Female	11 years	5.8 kg	Terminal
Mk-Li	2017_03_ FR	Male	12 years	7.6 kg	Terminal
Mk-Cs	2017_04E_ FR	Female	9 years	4.3 kg	Survival
Mk-Lo	2014_42E_ FR	Female	9 years	4.0 kg	Terminal
Mk-Sa	2017_04E_ FR	Female	7 years	4.4 kg	Survival
