# Supplementary material for: Recruitment of upper-limb motoneurons with epidural electrical stimulation of the cervical spinal cord
Source: Nat Commun. 2021 Jan 19;12:435. doi: 10.1038/s41467-020-20703-1 (PMC7815834; doi:10.1038/s41467-020-20703-1)
Supplement: Supplementary file 3 — Reporting Summary [file 41467_2020_20703_MOESM3_ESM.pdf]

nature research

Corresponding author(s): Marco Capogrosso, Nathan Greiner

Last updated by author(s): Feb 19, 2020

Reporting Summary

Nature Research wishes to improve the reproducibility of the work that we publish. This form provides structure for consistency and transparency in reporting. For further information on Nature Research policies, see Authors & Referees and the Editorial Policy Checklist .

Statistics

For all statistical analyses, confirm that the following items are present in the figure legend, table legend, main text, or Methods section.

n/a	Confirmed	
☐	☒	The exact sample size (n) for each experimental group/condition, given as a discrete number and unit of measurement
☐	☒	A statement on whether measurements were taken from distinct samples or whether the same sample was measured repeatedly
☐	☒	The statistical test(s) used AND whether they are one- or two-sided<br>*Only common tests should be described solely by name; describe more complex techniques in the Methods section.*
☐	☒	A description of all covariates tested
☐	☒	A description of any assumptions or corrections, such as tests of normality and adjustment for multiple comparisons
☐	☒	A full description of the statistical parameters including central tendency (e.g. means) or other basic estimates (e.g. regression coefficient) AND variation (e.g. standard deviation) or associated estimates of uncertainty (e.g. confidence intervals)
☐	☒	For null hypothesis testing, the test statistic (e.g. F, t, r) with confidence intervals, effect sizes, degrees of freedom and P value noted<br>*Give P values as exact values whenever suitable.*
☒	☐	For Bayesian analysis, information on the choice of priors and Markov chain Monte Carlo settings
☒	☐	For hierarchical and complex designs, identification of the appropriate level for tests and full reporting of outcomes
☒	☐	Estimates of effect sizes (e.g. Cohen's d, Pearson's r), indicating how they were calculated

Our web collection on statistics for biologists contains articles on many of the points above.

Software and code

Policy information about availability of computer code

Data collection	Data collection was performed with the following commercial and custom code:<br>- Custom code developed in RPvdsEx to control an RZ2 BioAmp Processor (Tucker-Davis Technologies, Alachua, US), to record the electromyographic activity induced by epidural electrical stimulation (EES) in monkeys.<br>- NIM Eclipse system software (Medtronic plc, Fridley, Minnesota, USA), to record the electromyographic activity induced by epidural electrical stimulation (EES) in humans.<br>The computer code to build the computational model, the simulation results, and the corresponding figures of the manuscript can be found at https://bitbucket.org/ngreiner/greiner_et_al_2020/src/master/.
Data analysis	Data analysis was performed using custom Matlab (Matlab, The Mathworks, Inc.) code.<br>Computer simulations were performed with COMSOL v5.2a (COMSOL, Burlington MA) and Matlab for the finite element modeling part, and with Python 3.7 and the NEURON simulation environment for the neurophysical simulations. The simulation results (and corresponding figures) presented in the article can be reproduced in full using the research material at https://bitbucket.org/ngreiner/greiner_et_al_2020/src/master/.

For manuscripts utilizing custom algorithms or software that are central to the research but not yet described in published literature, software must be made available to editors/reviewers. We strongly encourage code deposition in a community repository (e.g. GitHub). See the Nature Research guidelines for submitting code & software for further information.

Data

Policy information about availability of data

All manuscripts must include a data availability statement. This statement should provide the following information, where applicable:

- Accession codes, unique identifiers, or web links for publicly available datasets
- A list of figures that have associated raw data
- A description of any restrictions on data availability

All the routines and data necessary to reproduce the computational model and all figures related to simulations in this manuscript are accessible from the following public repository: https://bitbucket.org/ngreiner/greiner_et_al_2020/src/master/.
This repository also includes all the experimental data used in comparisons with simulation results. All the other experimental data can be delivered upon specific requests to the corresponding authors

Field-specific reporting

Please select the one below that is the best fit for your research. If you are not sure, read the appropriate sections before making your selection.

[x] Life sciences [ ] Behavioural & social sciences [ ] Ecological, evolutionary & environmental sciences

For a reference copy of the document with all sections, see nature.com/documents/nr-reporting-summary-flat.pdf

Life sciences study design

All studies must disclose on these points even when the disclosure is negative.

Sample size	No statistical methods were used to pre-determine sample sizes. All data was analyzed in all animals independently and no formal statistical comparison between populations was performed. Sample sizes are similar to those reported in previous publications using similar experimental procedures (Citation 39, Citation 55).
Data exclusions	The EMG signals recorded in one muscle of one animal were discarded from analysis: their interpretation led to aberrant conclusions.
Replication	To document the muscular responses induced in any of the 5 macaque monkeys involved in our experiments by any given epidural electrode and for any stimulation amplitude, we repeated the stimulation 4 times. Replication of the experimental protocol was successful in all animals. Replication of the entire protocols in a given animal on different days was not possible due to the experimental constraints described in the Methods of our manuscript (terminal procedures, or single acute surgical session).
Randomization	No randomization was introduced in our experiments. Since no formal statistical comparisons were required in our study and all animals were independently analyzed, no randomization was necessary and the same protocol was performed on all animals.
Blinding	The investigators were not blinded to tested conditions. All animals underwent the same protocol, therefore blinding is not relevant for this study.

Reporting for specific materials, systems and methods

We require information from authors about some types of materials, experimental systems and methods used in many studies. Here, indicate whether each material, system or method listed is relevant to your study. If you are not sure if a list item applies to your research, read the appropriate section before selecting a response.

Materials & experimental systems

n/a	Involved in the study	
[x]	[ ]	Antibodies
[x]	[ ]	Eukaryotic cell lines
[x]	[ ]	Palaeontology
[ ]	[x]	Animals and other organisms
[ ]	[x]	Human research participants
[x]	[ ]	Clinical data

Methods

n/a	Involved in the study	
[x]	[ ]	ChIP-seq
[x]	[ ]	Flow cytometry
[ ]	[x]	MRI-based neuroimaging

Animals and other organisms

Policy information about studies involving animals; ARRIVE guidelines recommended for reporting animal research

Laboratory animals	The study involved 5 Macaca Fascicularis monkeys.<br>4 of them were female, respectively aged of 11, 9, 9 and 7 years.<br>1 was male, aged of 12 years.

Wild animals	The study did not involve wild animals.
Field-collected samples	The study did not involve samples collected from the field.
Ethics oversight	All procedures were carried out in accordance to the Guide for Care and Use of Laboratory Animals and the principle of the 3Rs. Protocols were approved by local veterinary authorities of the Canton of Fribourg (authorizations are reported in the Methods of our manuscript) including the ethical assessment by the local (cantonal) Survey Committee on Animal Experimentation and acceptance by the Federal Veterinary Office (BVET, Bern, Switzerland). Authorization numbers for the experiments and each animal involved in the study are provided in Supplementary Table 2 of our manuscript.

Note that full information on the approval of the study protocol must also be provided in the manuscript.

Human research participants

Policy information about studies involving human research participants

Population characteristics	Data were acquired during routine clinical procedures. Data were anonymised and we don't have access to population characteristics .
Recruitment	Anonymized clinical data were provided to us by Dr. Etienne PRALONG. Data were acquired during routine clinical practice.
Ethics oversight	After discussion with SwissEthics we confirmed that the use of anonymised clinical data is not subject to further ethical oversight

Note that full information on the approval of the study protocol must also be provided in the manuscript.

Magnetic resonance imaging

Experimental design

Design type	Anatomical T1 and T2 weighted images of the cervical spine (not shown in the manuscript but available upon request) from animals sedated with ketamine.
Design specifications	GE 3T clinical research scanner.
Behavioral performance measures	No behavioral performance were measured.

Acquisition

Imaging type(s)	Structural.
Field strength	3
Sequence & imaging parameters	CT scans:: imaging type: SPIRAL / slice thickness: 2mm / pixel size: 0.33mm x 0.33mm.<br>MRI scans:: T1/T2 / slice thickness: 0.8mm / pixel size: 0.3125mm x 0.3125mm.
Area of acquisition	Brain and spinal cord.

Diffusion MRI ☐ Used ☒ Not used

Preprocessing

Preprocessing software	No pre-processing was performed. No functional data was acquired. Only structural anatomical measurements were performed using OsiriX DICOM reader, v 11.0
Normalization	N/A
Normalization template	N/A
Noise and artifact removal	N/A
Volume censoring	N/A

Statistical modeling & inference

Model type and settings	N/A
Effect(s) tested	N/A

Specify type of analysis: ☒ Whole brain ☐ ROI-based ☐ Both

Statistic type for inference (See Eklund et al. 2016)	N/A
Correction	N/A

Models & analysis

n/a	Involved in the study
☒	☐ Functional and/or effective connectivity
☒	☐ Graph analysis
☒	☐ Multivariate modeling or predictive analysis
